# Supplementary material for: Iron Related Biomarkers Predict Disease Severity in a Cohort of Portuguese Adult Patients during COVID-19 Acute Infection
Source: Viruses. 2021 Dec 10;13(12):2482. doi: 10.3390/v13122482 (PMC8703662; doi:10.3390/v13122482)
Supplement: Supplementary file 1 [file viruses-13-02482-s001.zip › viruses-1470773-supplementary.pdf]

Table S1 – Number of individual determinations per figure or table.

		COVID19 negative	COVID-19 positive	BD
	Iron	84	114	35
	Transferrin	84	114	35
	Tf sat	84	114	35
	Ferritin	84	114	35
	Hepcidin	89	96	35
	Heme	142	85	35
	Haptoglobin	23	33	10
	Erythropoetin	65	99	35
	RBC	169	124	
	Hb	169	124	
	HCT	169	124	
	MCV	169	124	
	MCH	169	124	
	MCHC	169	124	
	RDWCV	169	124	
	RDWSD	169	124	
	WBC	169	124	
	Neutrophils	155	122	
	Lymphocytes	155	122	
	Monocytes	155	122	
	platelets	169	124	
	platelets MPV	166	121	
	platelets PDW	166	121	
		Iron		
	severity 2	20	23	
	severity 3	40	44	
	severity 4	17	30	
	severity 5	5	17	
		Transferrin		
	severity 2	20	23	
	severity 3	40	44	
	severity 4	17	30	
	severity 5	5	17	
	Figure 1 and table 1	Ferritin		
Figures 2 and 3	severity 2	20	23	
	severity 3	40	44	
	severity 4	17	30	
	severity 5	5	17	
		WBC		
	severity 2	39	27	
	severity 3	67	47	
	severity 4	43	32	
	severity 5	6	18	
		Lymphocytes		
	severity 2	39	26	
	severity 3	62	47	
	severity 4	38	32	

Table S2– Distribution of COVID-19 patients among the severity groups, according to the presence of different comorbidities.

**Severity groups**	2	3	4	5	**Total**
Count	28	48	32	19	127
Percentage in each group	22.0	37.8	25.2	15.0	100
**Diabetes**					
Count	7	20	13	12	52
Percentage in each group	13.5	38.5	25	23.1	100
Percentage within each severity group	25.0	41.2	40.1	63.2	
**Severity groups**					
χ^2=6.883, p=0.077					
**Hypertension**					
Count	12	34	21	16	83
Percentage in each group	14.5	41.0	25.3	19.3	100
Percentage within each severity group	42.9	70.8	65.6	84.2	
χ^2=9.880, p=0.020					
**Obesity**					
Count	4	17	8	2	31
Percentage in each group	12.9	54.8	25.8	6.5	100
Percentage within each severity group	14.3	35.4	25.0	10.5	
χ^2=6.698, p=0.082					
**Dyslipidaemia**					
Count	7	21	15	13	56
Percentage in each group	12.5	37.5	26.8	23.2	100
Percentage within each severity group	25.0	43.4	46.9	13.0	
χ^2=8.805, p=0.032					
**CKD**					
Count	0	10	4	4	18
Percentage in each group	0	55.6	22.2	22.2	100
Percentage within each severity group	0	20.8	12.5	21.1	
χ^2=7.187, p=0.066					
**CRD**					
Count	5	8	2	4	18
Percentage in each group	27.8	44.4	11.1	16.7	100
Percentage within each severity group	17.9	16.7	6.3	21.1	
χ^2=2.225, p=0.522					
**Anemia**					
Count	0	4	0	0	4
Percentage in each group	0	100	0	0	100
Percentage within each severity group	0	8.3	0	0	
χ^2=6.797, p=0.079					
**Hypocoagulation**					
Count	1	8	2	0	11
Percentage in each group	9.1	72.7	18.2	0	100
Percentage within each severity group	3.6	16.7	6.3	0	
χ^2=6.842, p=0.077					

χ^2: Pearson Chi-Square, p: Asymptotic significance (two-sided)
